# Revision of the genus *Laophontodes* T. Scott (Copepoda, Harpacticoida, Ancorabolidae), including the description of a new species and a key to species

**DOI:** 10.3897/zookeys.997.56965

**Published:** 2020-11-25

**Authors:** Kai Horst George, Linda Maria Anne Lehmanski, Terue Cristina Kihara

**Affiliations:** 1 Senckenberg am Meer, German Centre for Marine Biodiversity Research DZMB, Südstrand 44, 26382, Wilhelmshaven, Germany; 2 University of Cologne, Biocenter, Zülpicher Str. 47b, 50674, Köln, Germany; 3 INES Integrated Environmental Solutions UG, c/o Senckenberg am Meer – German Centre for Marine Biodiversity Research DZMB, Südstrand 44, 26382, Wilhelmshaven, Germany

**Keywords:** Crustacea, Indian Ocean, Kairei Hydrothermal Vent Field, Laophontodinae, meiofauna, phylogeny, taxonomy

## Abstract

The description of *Laophontodes volkerlehmanskii***sp. nov.** (Copepoda, Harpacticoida, Laophontodinae Lang) from the deep sea of the Kairei Field, western Indian Ocean, prompted the examination of the phylogenetic status of *Laophontodes* T. Scott and the relationships within the genus. The allocation of *L. volkerlehmanskii***sp. nov.** to *Laophontodes* based on diagnostic characters was relatively straightforward, yet phylogenetic analysis of the genus considering 39 morphological characters detected not a single autapomorphy. This indicates that *Laophontodes*, which seems to form a monophylum with *Ancorabolina* George and *Bicorniphontodes* George, Glatzel & Schröder, actually represents the stem-lineage, retaining the characters of the common ancestor without having developed unique derived morphological characters. Most of the 13 known species of *Laophontodes* can be characterised by distinct apomorphies. However, phylogenetic comparison highlights some uncertainties due to the apparent heterogeneous distribution of some derived characters across the species, the weakness of other features, and the fragmentary and inadequate description of several species, which, in combination with the unavailability of type material, prevents a detailed comparison of several phylogenetically relevant characters. Thus, the analysis presented here provides a further step towards understanding the systematic relationships of and within *Laophontodes*, rather than a conclusive answer. Nonetheless, a detailed character discussion and a key to species are given.

## Introduction

Recent extensive revisions of the Ancorabolidae Sars, 1909 (e.g., George 2006; Gheerardyn and George 2010; Gheerardyn and Lee 2012; George and Müller 2013; George and Gheerardyn 2015; George 2017, 2018; George et al. 2019; Lee and Huys 2019; George 2020), revealed problems in the phylogenetic characterisation of the genus *Laophontodes* Sars, 1894 as a monophylum (cf. George and Gheerardyn 2015; George 2018, 2020; George et al. 2019); despite 13 species being assigned to *Laophontodes* (cf. Lee and Huys 2019; George 2020) not a single autapomorphy has been detected. Although genetic approaches can provide valuable information, molecular data for *Laophontodes* are not available, and morphological analyses are the most robust method for determining phylogenetic relationships. Such an approach must include the detailed descriptions of all species, including new ones, in order to enable the detection of not only species characteristics, but also derived features exclusively shared by all *Laophontodes* species (= synapomorphies), and thus facilitating phylogenetic comparisons (cf. George 2017). In that context, we describe a new *Laophontodes* species, *L. volkerlehmanskii* sp. nov. from the western Indian Ocean. It represents the first member of the genus in that geographic area, and one of few species inhabiting bathyal depths > 2000 m. Apart from a discussion on the systematics within *Laophontodes*, a key to the *Laophontodes* species is given.

## Material and methods

**Figure 1.**
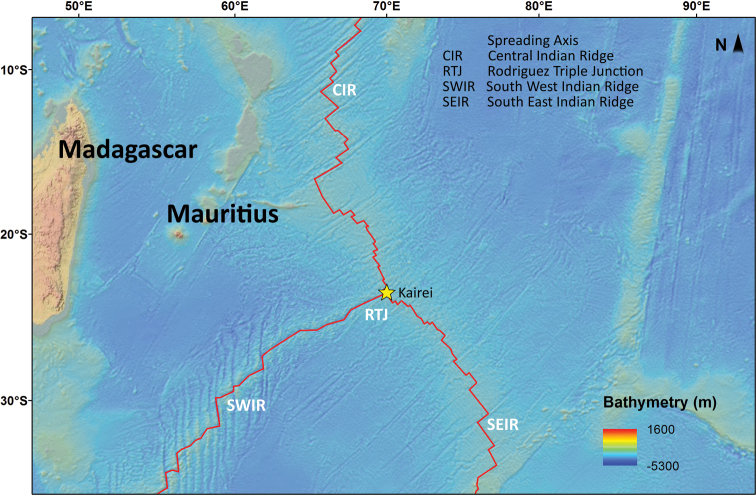
Map showing the place of discovery of the presented species. The star indicates the sampling station at the Kairei ridge, adapted from Dr Klaas Gerdes (Hamburg, Germany).

Samples were collected during the INDEX 2012 expedition of RV FUGRO GAUSS in December 2012 at the Kairei Field, an active hydrothermal vent field within the Central Indian Ridge (Kihara and Schröter 2013) (Fig. 1). Sampling was undertaken using a TV grab at a depth of 2467 m. The here described *Laophontodes volkerlehmanskii* sp. nov. was collected on 1 December, 2012 at station #I12_36T. The material collected was sieved with a 300 µm mesh and preserved in a solution of 4% buffered formalin, 96% ethanol, and DESS. Centrifugation with 40% Levasil and kaolin was performed three times at 5000 rpm for five minutes to extract the fauna. The individuals gathered were sorted by hand using a Leica M125 stereomicroscope.

Specimens to be studied were embedded in glycerol and put on slides for further investigation. Species identification and drawings were made with the use of a camera lucida on a Leica DMR compound microscope equipped with differential interference contrast optics.

Confocal Laser Scanning Microscopy (CLSM) was used to examine three individuals, two females and one male. The individuals were stained overnight with a 1:1 solution of Congo Red and Acid Fuchsin adapted from Michels and Büntzow (2010). Specimens were individually mounted in a drop of glycerine surrounded by a transparent, self-adhesive reinforcement ring to prevent direct contact between the specimen and coverslip and, therefore, damage to or distortion of the specimen. Images were generated using a Leica TCS SP5 consisting of a Leica DM5000 B upright microscope and three visible-light lasers. The software used was LAS AF 2.2.1 (Leica Application Suite Advanced Fluorescence). Images were taken with objective HCX PL APO CS 10.0× 0.40 DRY UV at an extinction wavelength of 561 nm with 80% acousto-optic tuneable filter. Using overlapping optical sections, passing through the whole specimen with an ideal number of sections determined by the software, a series of stacked images was generated. Table 1 lists the applied settings. To obtain a three-dimensional representation from selected body parts, the data produced during the CLSM scanning was processed with the software Drishti (http://anusf.anu.edu.au/Vizlab/drishti/). The obtained images were finalised with maximum projection and Adobe Photoshop CS6 for adjusting colour, contrast and brightness. The type material is kept in the collection of the Senckenberg Forschungsinstitut und Naturmuseum, Frankfurt am Main (Germany).

**Table 1.** Confocal laser scanning microscopy (CLSM) settings. Ch1 = detection channel 1.

Acquisition resolution	2048 × 2048
Numerical aperture	0.4
Excitation beam splitter	DD 488/561
Detected emission wavelength (nm)	Ch1: 570–629
	Ch2: 629–717
Detector gain	544 and 509 V
Amplitude offset	-1.7 and 0.8%
Pinhole aperture (µm)	53.0

The phylogenetic analysis strictly follows Hennig (1982) and Ax (1984, 1988, 1995) as explained by George (2020) and without the application of any computer-based cladistic programs. Consequently, Fig. 11 is not a computer-generated cladogram; instead, it is a manually generated clear presentation of the results of the phylogenetic discussion.

General terminology follows Lang (1948), Huys and Boxshall (1991), and Huys et al. (1996). Terminology referring to phylogenetic aspects follows Ax (1984); the terms “telson” and “furca” are adopted from Schminke (1976).

Abbreviations used in the text:

**A1**: antennule;

**A2**: antenna;

**aes**: aesthetasc;

**cphth**: cephalothorax;

**enp-1–enp-3**: endopodal segments 1–3;

**exp-1–exp-3**: exopodal segments 1–3;

**FR**: furcal rami;

**GDS**: genital double somite;

**GF**: genital field;

**md**: mandible;

**mx**: maxilla;

**mxl**: maxillule;

**mxp**: maxilliped;

**n**: number of specimens

**P1–P6**: swimming legs 1–6;

**R**: rostrum;

**STE**: subapical tubular extension.

## Results

### Subclass Copepoda Milne Edwards, 1840

**Order Harpacticoida Sars, 1903**

**Family Ancorabolidae Sars, 1909**

**Subfamily Laophontodinae Lang, 1944**

#### Genus Laophontodes

Taxon classificationAnimaliaHarpacticoidaAncorabolidae

T. Scott, 1894

93972C54-7DDA-574D-A5CD-CDCD99F9A286

##### Species composition.

*L. typicus* T. Scott, 1894 (type species); *L. antarcticus* Brady, 1918, *L. georgei* Lee & Huys, 2019, *L. gertraudae* George, 2018, *L. macclintocki* Schizas & Shirley, 1994, *L. monsmaris* George, 2018, *L. mourois* Arroyo, George, Benito & Maldonado, 2003, *L. sabinegeorgeae* George & Gheerardyn, 2015, *L. sarsi* George, 2018, *L. scottorum* George, 2018, *L. spongiosus* Schizas & Shirley, 1994, *L. whitsoni* T. Scott, 1912 (cf. George 2020); *species inquirenda*: *L. propinquus* Brady, 1910.

##### Remarks.

Lee and Huys (2019) listed 18 species in *Laophontodes* – 15 valid species plus one *species incertae sedis* (*L. propinquus* Brady, 1910) and two *species inquirendae* (*L. antarcticus* Brady, 1918, *L. ornatus* Krishnaswamy, 1957). The number of species was updated by George et al. (2019), who established the genus *Bicorniphontodes* George, Glatzel & Schröder, 2019 to include the then newly described *B. clarae* George, Glatzel & Schröder, 2019, along with *Laophontodes bicornis* A. Scott, 1896, *L. hamatus* (Thomson, 1883), *L. horstgeorgei* George & Gheerardyn, 2015, and *L. ornatus* Krishnaswamy, 1957. George et al. (2019) reduced the number of species allocated to *Laophontodes* to 14. Of these, *Laophontodes brevis* Nicholls, 1944 was excluded from the current analysis: although Lee and Huys (2019: 367) are certainly right when insisting on its validity as a species, since Lang (1965) did not formally synonymise *L. brevis* with *L. bicornis* (now *Bicorniphontodes bicornis*), the remarkable similarity of *L. brevis* with *B. bicornis* noted by Lang (1965) clearly points to its affiliation to *Bicorniphontodes* instead to *Laophontodes*. Therefore, we follow the list of 13 species of *Laophontodes* as provided by George (2020).

#### Laophontodes volkerlehmanskii sp. nov.

Taxon classificationAnimaliaHarpacticoidaAncorabolidae

029F3C48-842F-5B8D-BCD4-D552146FE41B

http://zoobank.org/34839233-C919-4892-9CA9-8FAFEFCF0D70

Figs 2 , 3 , 4 , 5 , 6 , 7 , 8 , 9 , 10

##### Locus typicus.

Indian Ocean, Central Indian Ridge, Kairei Field, station #I12_36T, geographic position 25°19.240'S, 70°02.433'E, 2467 m depth.

##### Type material.

Four females and four males collected during research cruise INDEX 2012 on December 1^st^, 2012. Holotype: female, not dissected, on one slide, collection number SMF 37216/1; paratype 1 (allotype): male, not dissected, on one slide, collection number SMF 37217/1; paratype 2: female, dissected and mounted onto 15 slides, collection number SMF 37218/1–15; paratype 3: male, dissected and mounted onto two slides, collection number SMF 37219/1–2; paratype 4: male, not dissected, on one slide, collection number SMF 37220/1; paratype 5: male, not dissected, on one slide, collection number SMF 37221/1; paratype 6: female, not dissected, on one slide, collection number SMF 37222/1; paratype 7: female, not dissected, on one slide, collection number SMF 37223/1.

##### Description.

**Figure 2.**
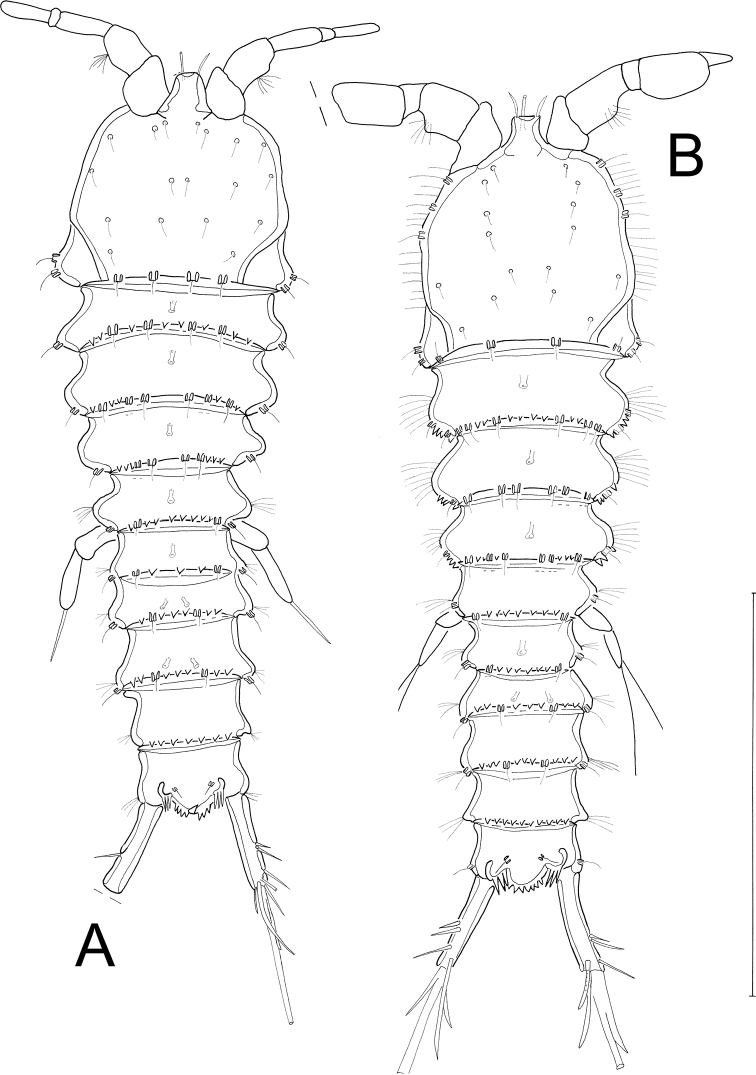
*Laophontodes volkerlehmanskii* sp. nov. **A** female holotype (SMF 37216/1), habitus, dorsal view **B** male paratype 1 (allotype) (SMF 37217/1), habitus, dorsal view. Scale bar 200 µm.

**Figure 3.**
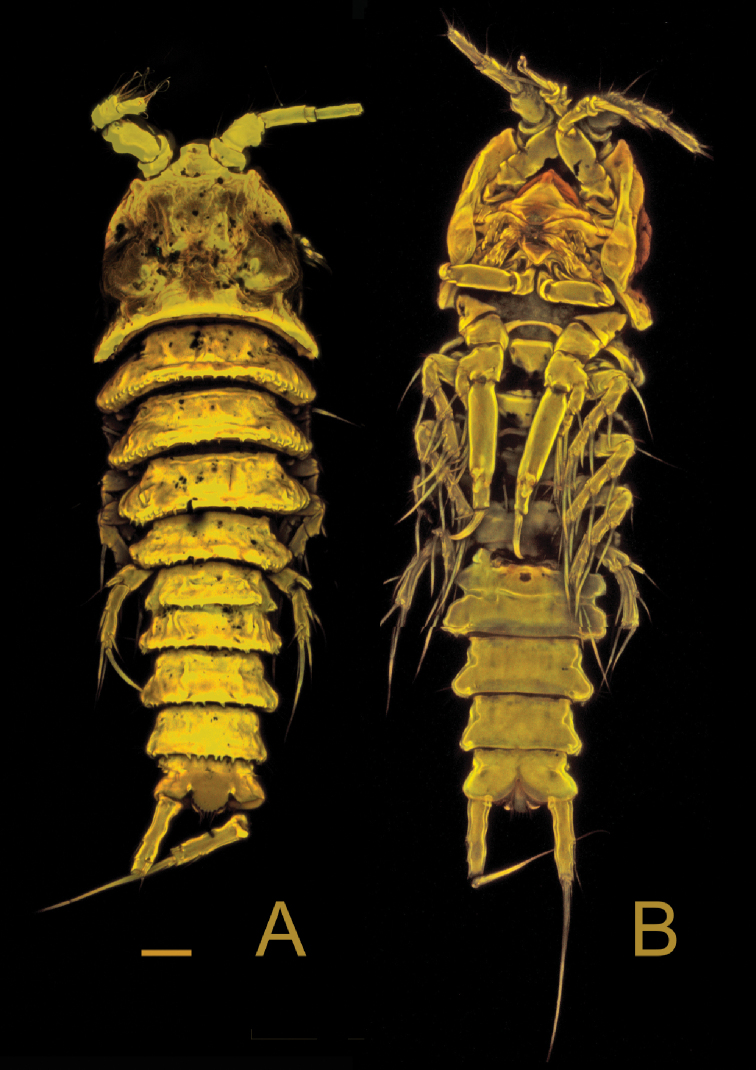
*Laophontodes volkerlehmanskii* sp. nov. Confocal laser scanning microscopy images of **A** female paratype 6 (SMF 37222/1), habitus, dorsal view **B** female paratype 7 (SMF 37223/1), habitus, ventral view. Scale bar: 25 µm.

**Figure 4.**
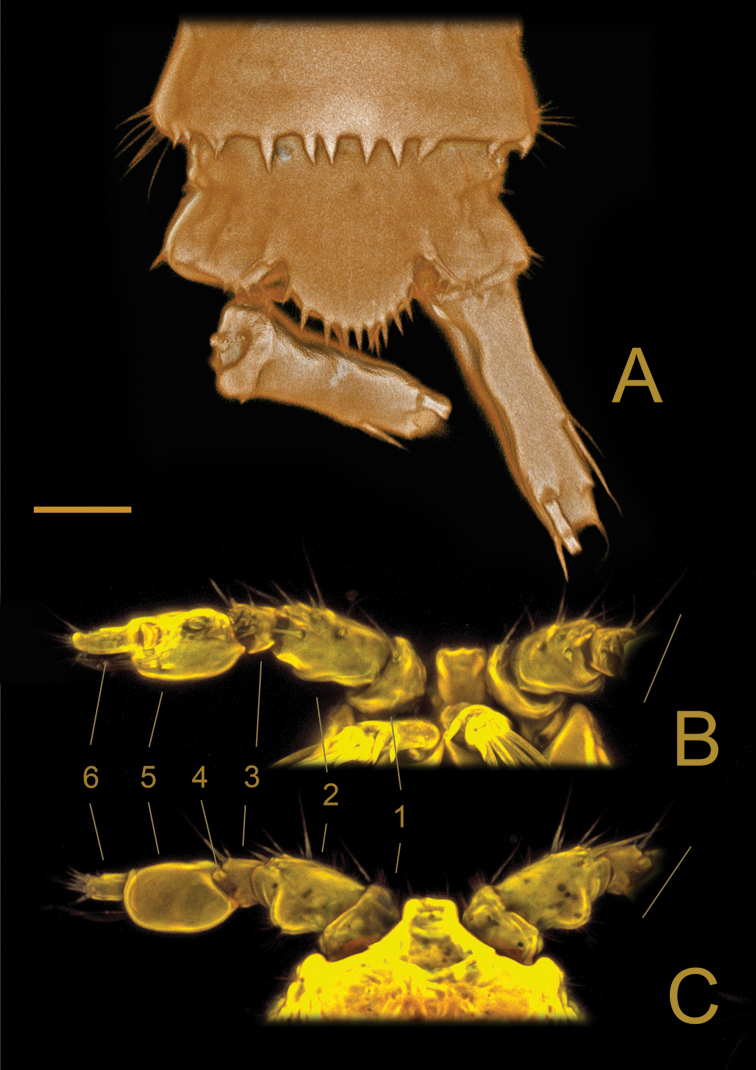
*Laophontodes volkerlehmanskii* sp. nov. Three-dimensional representation (Drishti software) based on confocal laser scanning microscopy images of Female paratype 6 (SMF 37222/1) **A** anal operculum, dorsal view; Confocal laser scanning microscopy images of male paratype 4 (SMF 37220/1) **B**A1, ventral view **C** male paratype 4 (SMF 37220/1), A1, dorsal view, numbers refer to antennular segments Scale bar: 400 µm (**A**); 25 µm (**B, C**).

**Figure 5.**
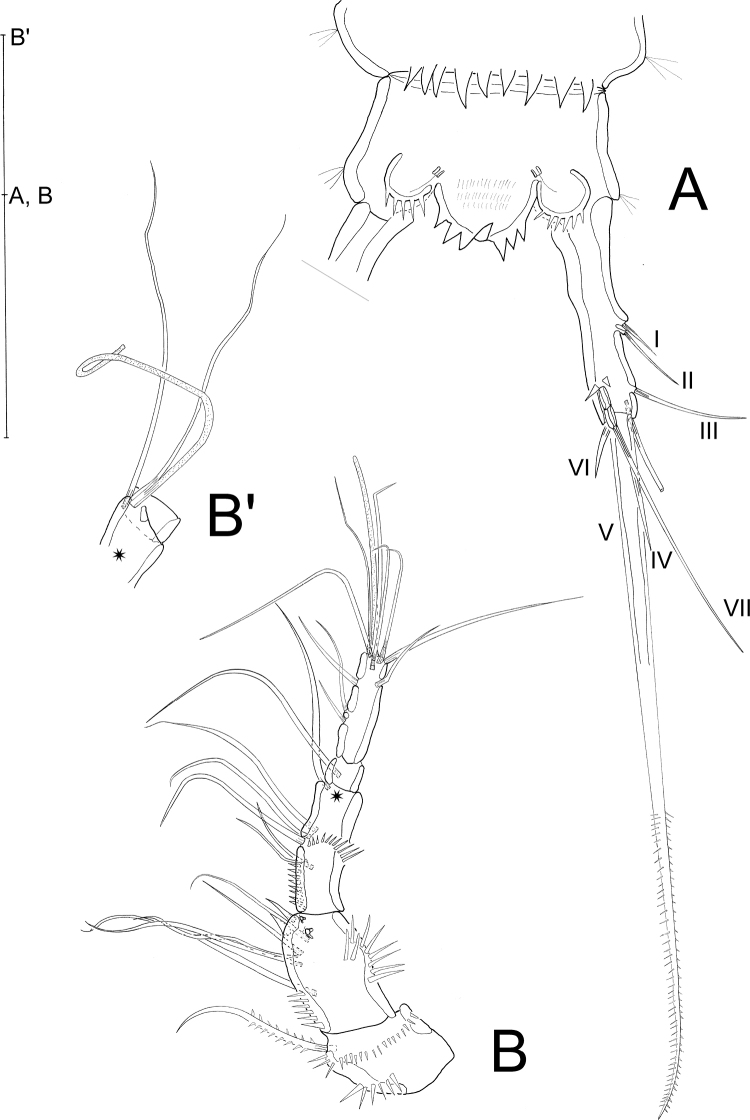
*Laophontodes volkerlehmanskii* sp. nov., female holotype (SMF 37216/1) **A** telson and right furcal ramus, dorsal view; Roman numbers indicate furcal setae, **B**A1, **B**' Posterior margin of fourth antennular segment, showing projection that bears the acrothek. Scale bar: 50 µm.

**Female**: Habitus (Figs 2A, 3A, B) cylindrical, body length (R to end of FR) (median value) = 399 µm (390–405 µm; *N* = 3). R small, fused to cphth, with 2 sensilla (one sensillum missing in Fig. 2A) and 1 apical tube pore. Cphth reaching more than 25% of total body length, with posterior swelling on each side; dorsally covered by sensilla, those on posterior margin arising from socles. Body somites clearly distinct. Last thoracic and first abdominal somites fused forming the GDS, juncture seen as dorsal serration. Posterior margins of free body somites, excluding telson, serrated dorsally, and with sensilla arising from small socles; P2–P5-bearing somites additionally with 1 dorsal tube pore centrally; P6 and P7-bearing somites carrying 2 dorsal tube pores centrally. Telson (Figs 2A, 3A, B, 4A) slightly smaller than preceding somite, with FR set widely apart. Anal operculum (Figs 4A, 5A) with distinct, strong apical spinules; basally with pair of sensilla and additional spines above bases of FR.

FR (Figs 4A, 5A) slender, about 4 times as long as wide, with distal tube pore and 7 bare setae: seta II dorsal to, and twice as long as I; III subapical; IV and V apical and fused at base, V very long and narrow, distal 1/3 bipinnate; VI apical on inner margin, bare and short; VII dorsal, tri-articulated.

A1 (Fig. 5B, B') 5-segmented. First segment carrying 1 bipinnate seta and 2 rows of spinules on apical edge below seta; second segment with 9 setae (2 setae broken in Fig. 5B) and 1 row of spinules each on outer and inner margin; third segment with 7 bare setae and 1 aes (fused to 1 seta) (Fig. 5B'); fourth segment partly overlapped by preceding one (Fig. 5B'), with 1 bare seta; fifth segment with 10 bare setae, 2 of which forming an apical trithek with1 aes. Setal formula: 1-1/2-9/3-6+(1+aes)/4-1/5-8+(2+aes).

**Figure 6.**
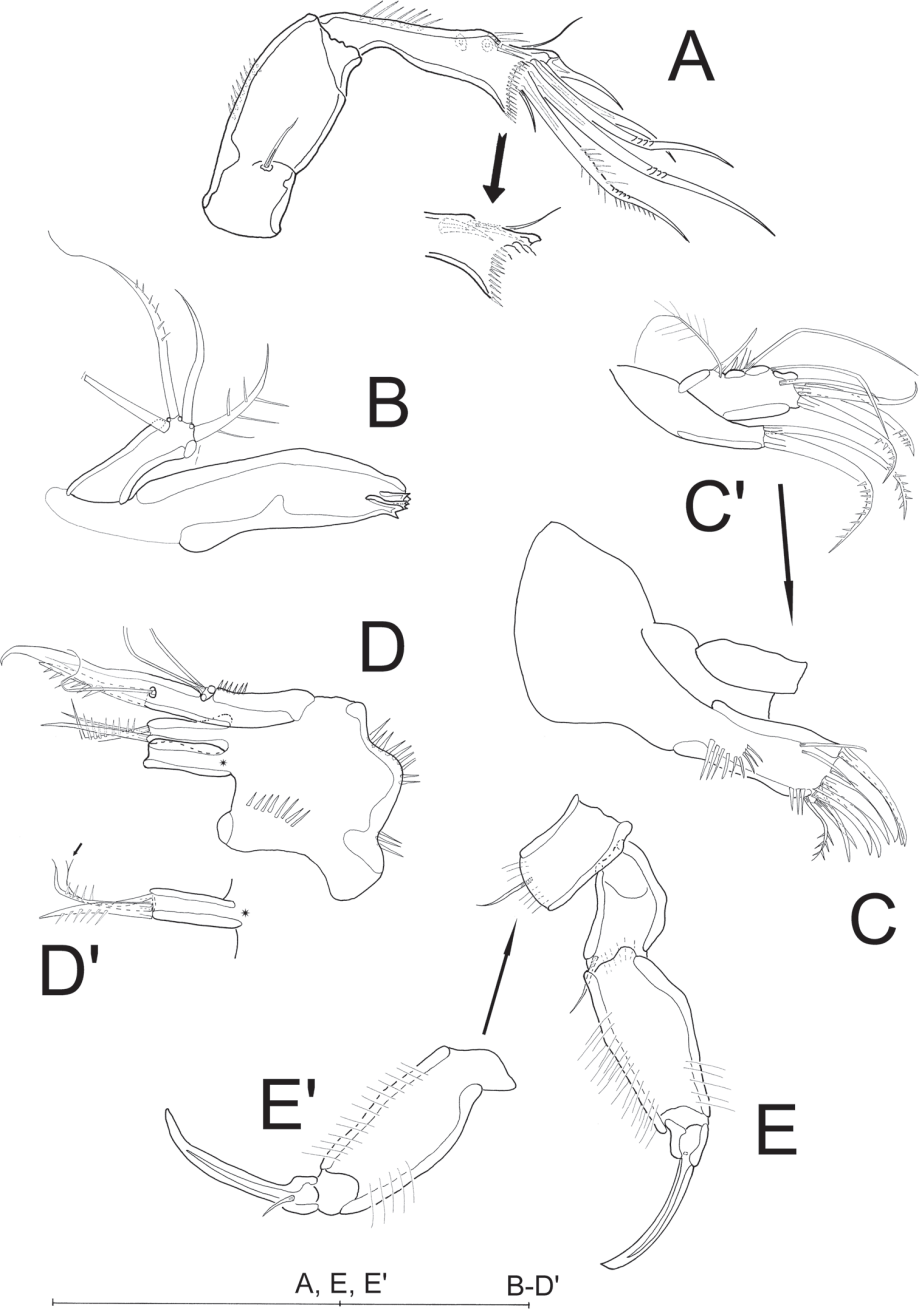
*Laophontodes volkerlehmanskii* sp. nov., female paratype 2 (SMF 37218/1–15) **A**A2**B** Md **C** Mxl gnathobase **C**' Mxl coxa and basis **D** Mx **D**' Mx, proximal endite; arrow pointing to cleft tip **E** Mxp **E**' Mxp (counterpart), showing minute accompanying seta. Scale bar: 50 µm.

A2 (Fig. 6A). Allobasis without abexopodal seta. Exopod represented by minute bare seta. Endopod with 2 rows of inner spinules – 1 subapical and 1 proximal – ; additionally, with 2 bare spines and 1 fine bare seta accompanied by 4 anterior spinules; apically with 5 setae, of which 3 geniculated and 1 biplumose.

Md (Fig. 6B) with slender gnathobase bearing 4 teeth; palp 1-segmented, with 5 setae (1 missing, and 1 damaged in Fig. 6B), of which 1 biplumose and 1 unipinnate.

Mxl (Fig. 6C, C'). Praecoxal arthrite (Fig. 6C) with 1 row of spinules basally, 8 bare apical spines and 1 subapical biplumose seta, additionally with 2 surface setae; coxa (Fig. 6C') with 2 bipinnate apical setae; basis, endopod and exopod fused to single lobe (Fig. 6C') carrying 2 unipinnate and 1 bare apical seta, 1 bare subapical seta, 3 bare and 1 bipinnate seta and few outer spinules.

Mx (Fig. 6D, D'). Syncoxa bearing 3 rows of spinules and 2 endites. Proximal endite with 2 plumose setae and 1 bare seta with cleft tip (arrow in Fig. 6D'), distal endite with 1 plumose and 2 bare setae. Allobasis distinct, terminally with strong claw accompanied by 1 plumose and 1 fine, bare seta. Endopod 1-segmented, knob-like, with 2 bare setae.

Mxp (Fig. 6E, E') prehensile; syncoxa bearing 1 bare seta and single row of spinules apically (Fig. 6E); basis with 1 row of spinules on inner and outer margins; endopod drawn out into strong claw basally accompanied by minute, bare seta (Fig. 6E').

**Figure 7.**
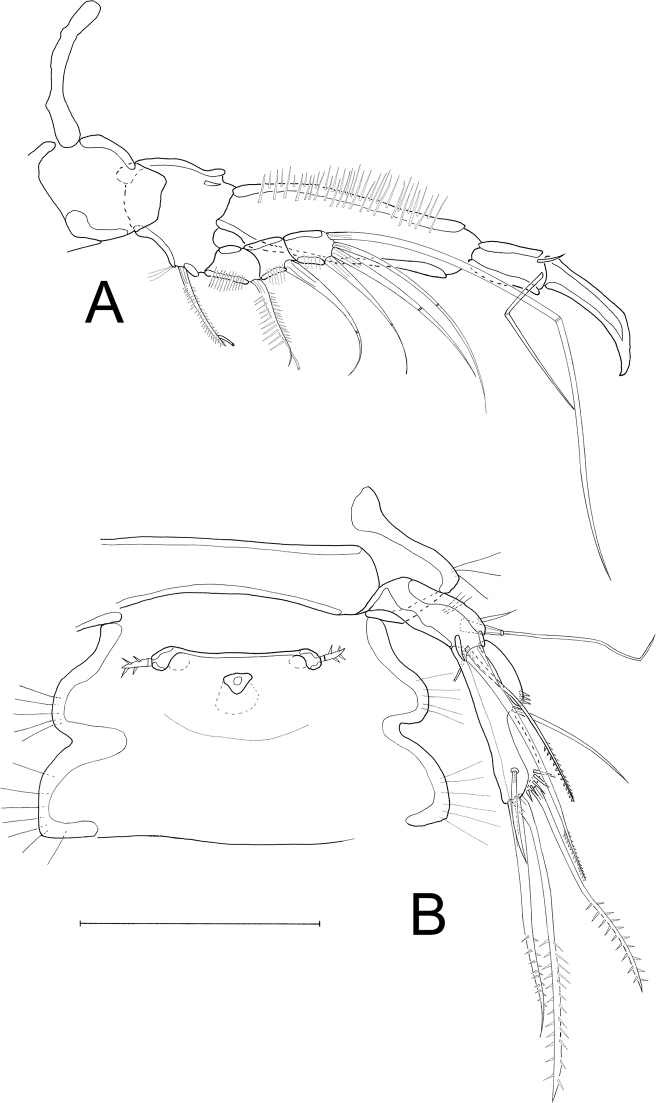
*Laophontodes volkerlehmanskii* sp. nov., female paratype 2 (SMF 37218/1–15) **A**P1**B**P5 and GF with minute P6. Scale bar: 50 µm.

P1 (Figs 3B, 7A) with slender and bow-like intercoxal sclerite, and large, triangular praecoxa (Fig. 3B); coxa and basis with slight longitudinal elongation, outer margin of basis forming pedestal for exopod, with 1 biplumose outer seta carrying STE, and 1 minute anterior inner seta. Endopod 2-segmented, enp-1 strong and elongate, with 2 rows of spinules on inner margin; enp-2 small, approximately 1/3 the length of enp-1, apically with 1 strong claw, 1 long, slender, geniculated seta, and 1 minute seta. Exopod 3-segmented, less than half the length of endopod, each segment with outer spinules, exp-1 carrying 1 biplumose outer seta with STE; 2 with 1 bare geniculated outer seta; exp-3 apically with 4 bare, geniculated setae.

**Figure 8.**
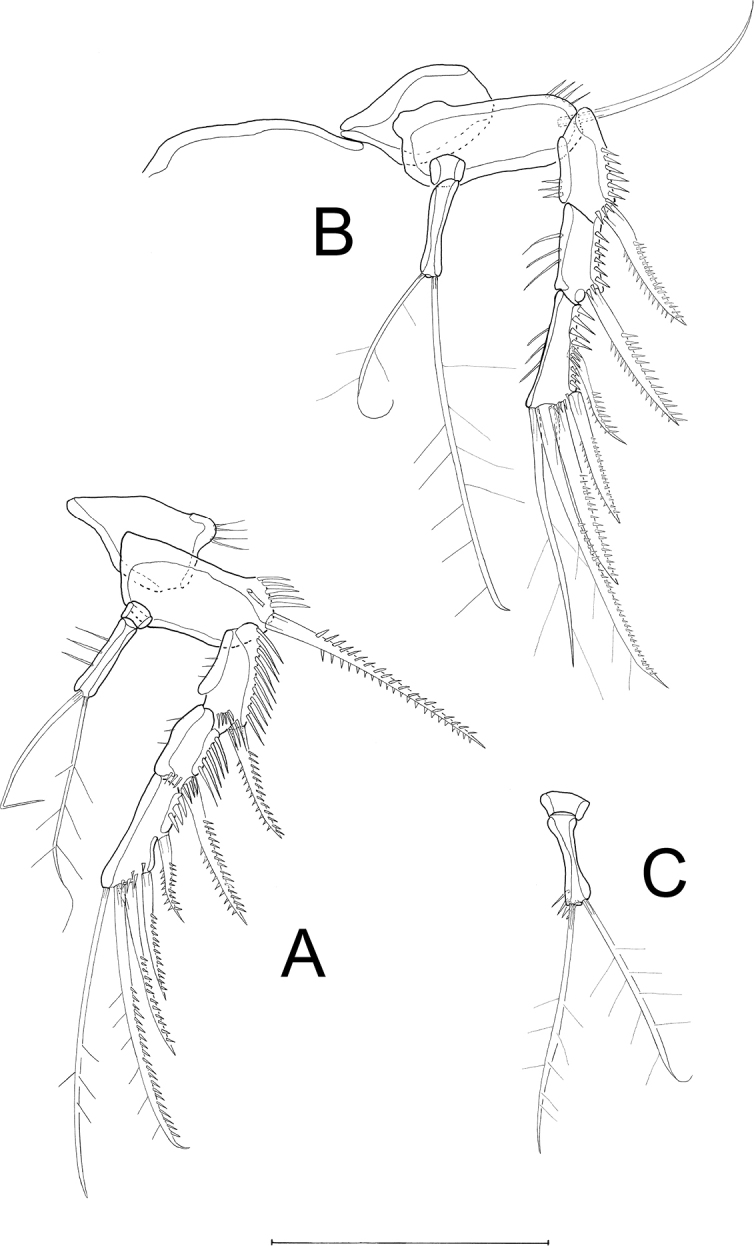
*Laophontodes volkerlehmanskii* sp. nov., female paratype 2 (SMF 37218/1–15) **A**P2**B**P3**C**P4 endopod. Scale bar: 50 µm.

P2–P4 (Fig. 8A–C) with transversely elongated bases with outer margin bearing 1 long seta, the latter bipinnate in P2, bare in P3 and P4; exopods 3-segmented, endopods 2-segmented. All exopodal segments with outer row of robust spinules, and fine inner spinules. Exp-1 and exp-2 with 1 bipinnate outer spine; exp-3 with 3 pinnate outer spines, apically with 1 spine, whose inner margin plumose and outer margin pinnate, and 1 slender biplumose seta. Endopods narrow, enp-1 small, without spinules or setae; enp-2 elongate, of P2 and P4 with spinules, P2–P4 with 2 apical setae, both biplumose in P3 and P4, inner apical seta bare in P2. See Table 2 for setal formula.

P5 (Fig. 7B) with short setophore on slender baseoendopod carrying 1 long bare seta and a few spinules; endopodal lobe reduced, represented by 2 bipinnate setae. Exopod fused to baseoendopod, slender, with 1 bare outer seta, 1 bare seta displaced to anterior surface, and 3 plumose setae – 1 subapical and 2 apical.

GF (Fig. 7B) with single gonopore. P6 strongly reduced, limbs fused into single small plate, with pair of minute bipinnate spines.

**Male**: The male differs from the female in the following characters: habitus, A1, P3 and P4 endopod, and P5.

**Figure 9.**
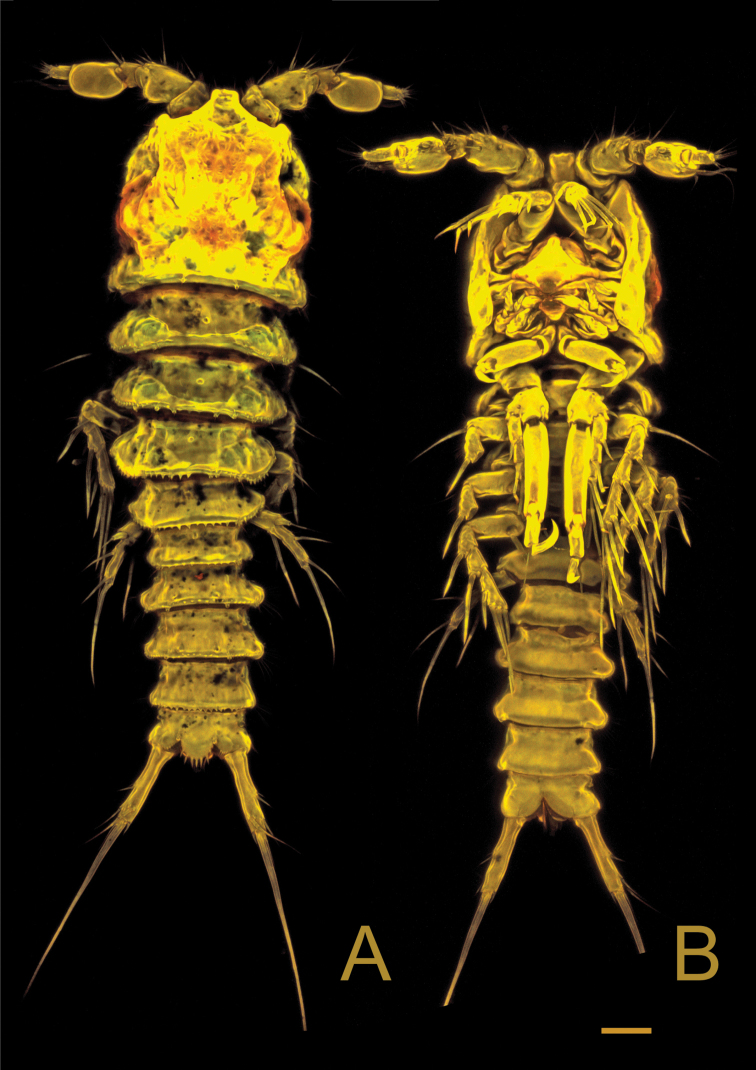
*Laophontodes volkerlehmanskii* sp. nov. Confocal laser scanning microscopy images of male paratype 4 (SMF 37220/1) **A** habitus, dorsal view **B** habitus, ventral view. Scale bar: 25 µm.

Habitus (Figs 2B, 9) as in female, but slightly longer, body length (from R to FR) (median value) = 402 µm (378–426 µm; *N* = 2); cphth with more dorsal sensilla than female; with antero-lateral sensilla arising from socles.

**Figure 10.**
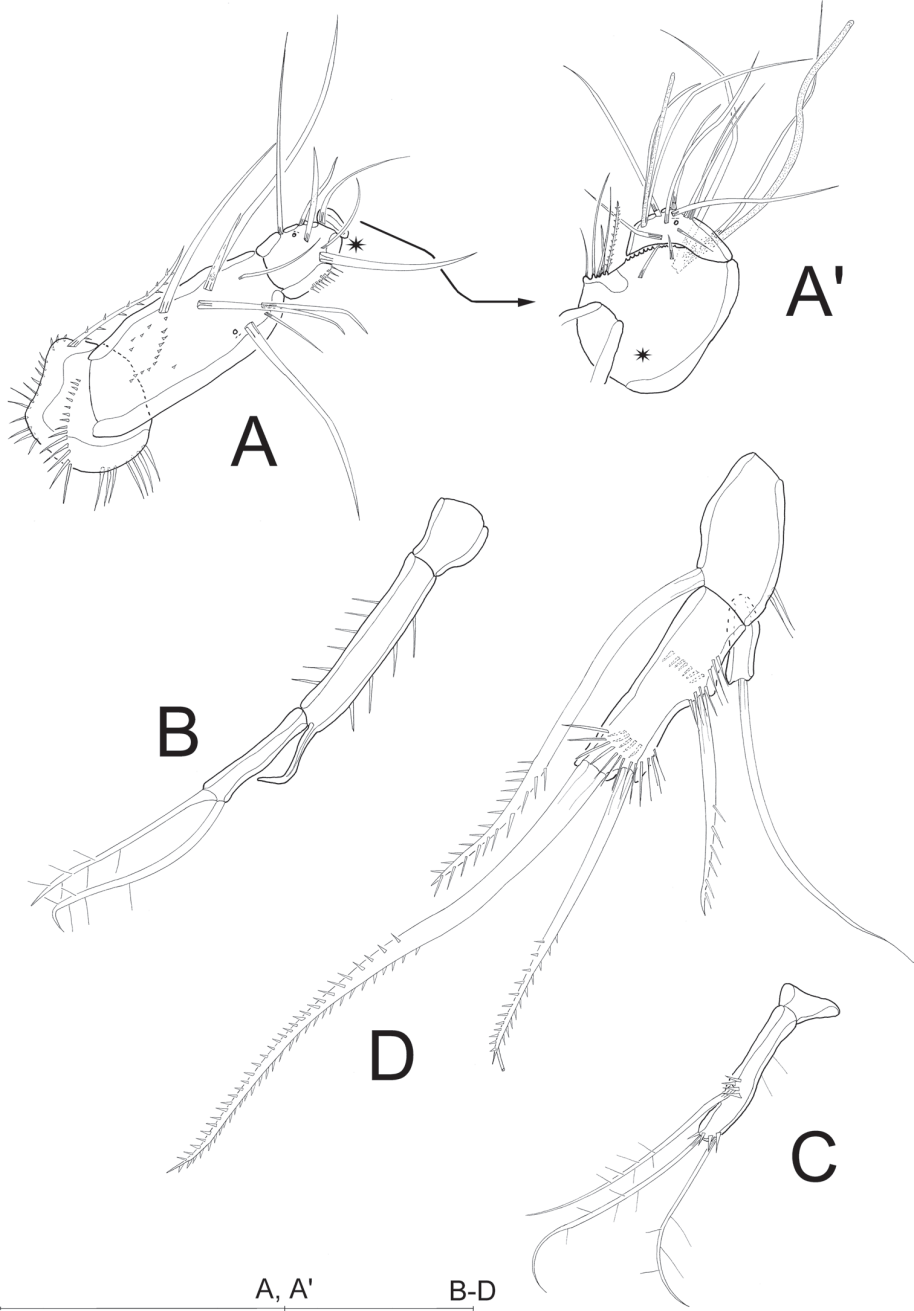
*Laophontodes volkerlehmanskii* sp. nov., male paratype 3 (SMF 37219/1–2) **A**A1**A**' fifth and sixth antennular segment **B**P3 endopod **C**P4 endopod **D**P5. Scale bar: 50 µm.

A1 (Figs 4B, C, 10A, A') 6-segmented, chirocer; first segment with 1 bipinnate seta and 3 rows of spinules; second segment with 9 bare setae (one seta missing in Fig. 10A), and a row of short spinules; third segment with 6 bare setae (one seta missing in Fig. 10A), and single row of spinules, segment partially overlapping fourth and fifth segment; fourth segment (Fig. 4C, * in Fig. 10A) minute, almost completely covered by fifth segment, with 1 bare seta; fifth segment (Fig. 10A') swollen, with 9 setae (1 biplumose, 8 bare), 2 of which form an acrothek with 1 aes, cuticle thorn-like at upper margin; sixth segment with 10 bare setae (1 seta missing in Fig. 10A'), 2 of which form an acrothek with 1 small aes. Setal formula: 1-1/2-8/3-6/4-1/5-7+(2+aes)/6-8+(2+aes).

P3 exopod as in female, endopod (Fig. 10B) 3-segmented; enp-1 minute and unarmed; enp-2 longest, with rows of spinules on inner and outer margins, lacking setae but inner apical margin with curved apophysis reaching to 2/3 the length of enp-3; enp-3 about 2/3 the length of enp-2, with 2 biplumose apical setae.

P4 exopod as in female, endopod (Fig. 10C) 2-segmented; enp-2 with 1 additional flexible outer spine accompanied by few spinules at its base; apically with 2 biplumose setae. The setal formula for P3 and P4 is given in Table 2.

**Table 2.** *Laophontodes volkerlehmanskii* sp. nov., setation of P2–P4. Roman numerals indicate outer spines.

	Exp-1	Exp-2	Exp-3	Enp-1	Enp-2	Enp-3
P2	I-0	I-0	III-2-0	0	0-2-0	–
P3 female	I-0	I-0	III-2-0	0	0-2-0	–
P3 male	I-0	I-0	III-2-0	0	0 (apophysis)	0-2-0
P4 female	I-0	I-0	III-2-0	0	0-2-0	–
P4 male	I-0	I-0	III-2-0	0	I-2-0	–

P5 (Fig. 10D) baseoendopod longer than broad, with 1 outer seta arising from short setophore; endopodal lobe incorporated into basal part of baseoendopod and represented by 1 long seta with bipinnate distal half; exopod not fused to baseoendopod, with 1 outer unipinnate seta, and 2 bipinnate setae – 1 subapical and bearing STE, and 1 apical.

##### Etymology.

The epithet *volkerlehmanskii* is given in dedication to the 60^th^ birthday of LMA Lehmanski’s father Volker Lehmanski (Gelsenkirchen, Germany).

**Figure 11.**
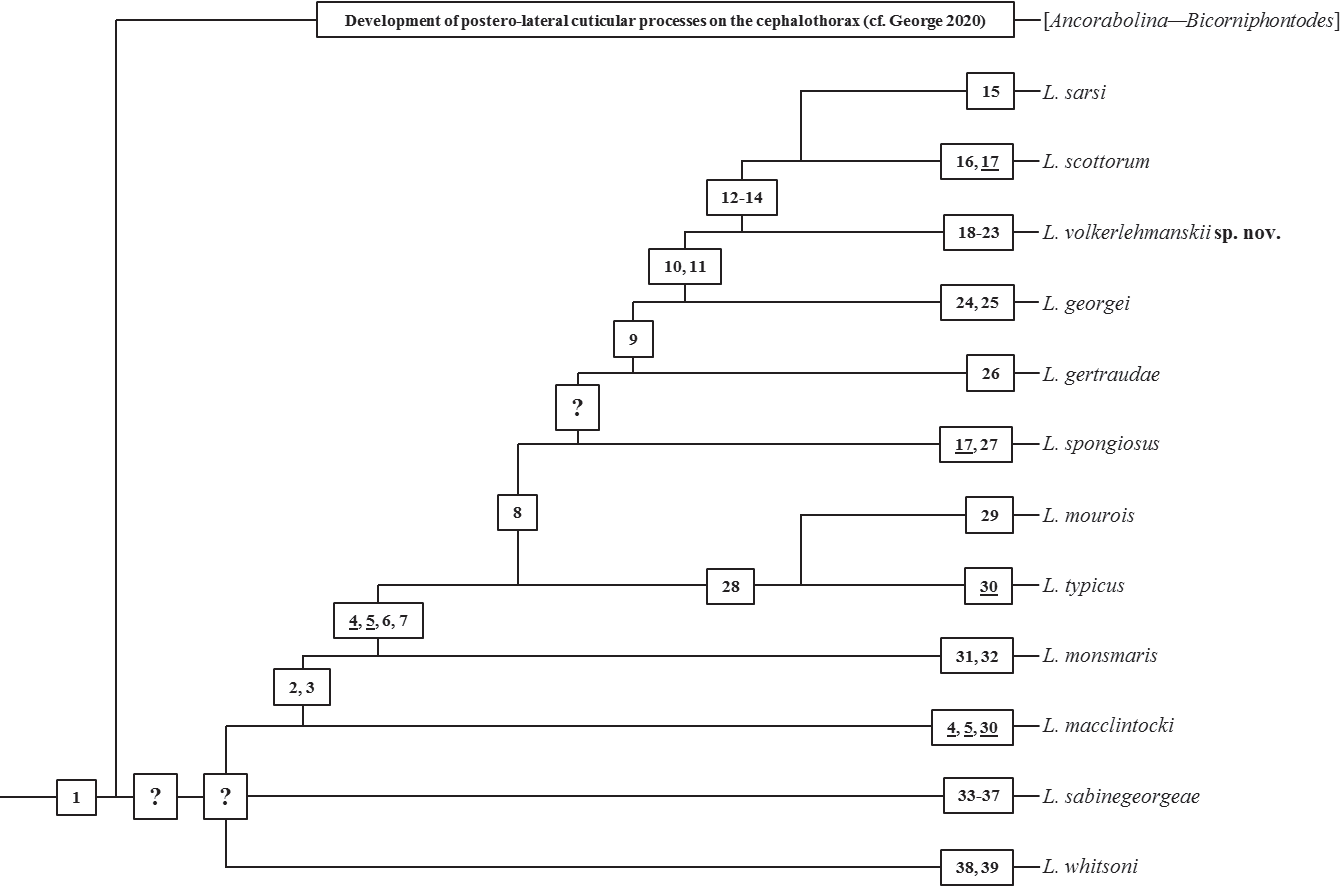
Cladogram summarizing the results of the phylogenetic analysis provided in the present contribution. Numbers in rectangles pointing to discussed characters listed in Table 3. Underlined numbers refer to convergent deviations. Detailed explanations are given in the text.

#### Diagnostic key to the species of Laophontodes^*^

**Table:** 

1	Body slender, cylindrical; cphth about 1/4^th^ of total body length (incl. FR)	**2**
–	Body compact, partially compressed dorsoventrally; cphth about 1/3^rd^ of total body length (incl. FR)	***Laophontodes scottorum* George, 2018**
2	Second antennular segment with flat posterior surface	**3**
–	Second antennular segment with posterior surface produced into bump	**4**
3	Mxp of moderate size; P3 and P4exp-3 with 1 inner seta; telson not overlapped by preceding somite dorsally; male antennule subchirocer	***Laophontodes macclintocki* Schizas & Shirley, 1994**
–	Mxp extremely strengthened; P3 and P4exp-3 with 2 inner setae; telson overlapped by preceding somite; male antennule chirocer	***Laophontodes monsmaris* George, 2018**
4	Apical claw on P1enp-2 narrow and thin; male antennule subchirocer	**5**
–	Apical claw on P1enp-2 wide and thickened; male antennule chirocer or subchirocer	**6**
5	Pedigerous and abdominal somites dorsally with fine ripples; P2–P4exp-3 with 1:2:2 inner setae	***Laophontodes whitsoni* T. Scott, 1912**
–	No ripples on body somites, abdominal somites except telson dorsally with H-like cuticular structures; P2–P4 with 0:0:1 inner setae	***Laophontodes spongiosus* Schizas & Shirley, 1994**
6	P2 and P3 exp-2 with 1 inner seta, P3 and P4exp-3 with 2 inner setae; rostrum with setulose tuft frontally; pedigerous and abdominal somites with fine ripples dorsally; abdominal somites except telson with paired cuticular ridges dorsally	***Laophontodes sabinegeorgeae* George, 2018**
–	These characters not combined	**7**
7	P4exp-3 inner apical seta short, flagelliform, bare	**8**
–	P4exp-3 inner apical seta long, biplumose	**10**
8	P2 and P3exp-3 inner apical seta short, flagelliform, bare	***Laophontodes sarsi* George, 2018**
–	P2 and P3exp-3 inner apical seta long, biplumose	**9**
9	P2–P4exp-3 without inner setae; P4 endopod 1-segmented; inner margin of male P3enp-3 straight	***Laophontodes georgei* Lee & Huys, 2019**
–	P2–P4exp-3 with 1 inner seta; P4 endopod 2-segmented; inner margin of male P3enp-3 bulged out	***Laophontodes mourois* Arroyo, George, Benito & Maldonado, 2003**
10	P1exp-1 outer spine unipinnate, comb-shaped, with strong outer pinnae; anal operculum with row of fine spinules on apical margin	**11**
–	P1exp-1 outer spine bipinnate, of normal shape; anal operculum with few strong spinules on apical margin	***Laophontodes volkerlehmanskii* sp. nov.**
11	P2–P4-bearing somites with fine ripples dorsally; maxillipedal claw without accompanying minute seta; female P5 baseoendopodal inner seta fish-bone-like	***Laophontodes typicus* T. Scott, 1894**
–	P2–P4-bearing somites without ripples; maxillipedal claw with accompanying minute seta; female P5 baseoendopodal inner seta bipinnate, of normal shape	***Laophontodes gertraudae* George, 2018**

## Discussion

### *Laophontodes* as “survivor” of the stem-lineage

The type genus of the Laophontodinae – *Laophontodes* – is the only representative of that subfamily which cannot be characterised by autapomorphies (cf. George and Gheerardyn 2015; George 2018, 2020). This causes certain complications, especially as *Laophontodes* comprises 13 species (cf. George 2020), plus the herein described *L. volkerlehmanskii* sp. nov.. As discussed by George (2020), *Laophontodes* seems to be closely related to *Ancorabolina* George, 2006 and *Bicorniphontodes* George, Glatzel & Schröder, 2019. These three genera presumably originate from a common ancestor, which developed one derived, apomorphic, feature (character 1 in Table 3 and below) [supposed ancestral, plesiomorphic, state in square brackets]:

1. A2 exopod lost and represented by 1 tiny seta only [A2 exopod 1-segmented, at least knob-like].

For a detailed discussion of character 1, see George (2020). Since all representatives of the three named genera share that apomorphy, George (2020) interpreted it as autapomorphic for the monophylum [*Ancorabolina – Bicorniphontodes – Laophontodes*] (Fig. 11). *Ancorabolina* and *Bicorniphontodes* share one further apomorphy, viz. the postero-lateral cuticular processes on the cephalothorax and thus form sister-groups, but are otherwise characterised by distinct autapomorphies (George 2020). However, this is not the case for *Laophontodes*. Species can only be assigned to *Laophontodes* using diagnostic characters and this resulted in *Laophontodes* becoming a conglomeration of many, at least partly, unrelated species. Consequently, several authors have excluded species from *Laophontodes*, placing them in newly erected and phylogenetically well-justified distinct genera (e.g., Lang 1965; George 2017: *Paralaophontodes* Lang, 1965; Conroy-Dalton 2004: *Lobopleura* Conroy-Dalton, 2004; Gheerardyn and Lee 2012: *Calypsophontodes* Gheerardyn & Lee, 2012; George et al. 2019: *Bicorniphontodes*; Lee and Huys 2019: *Rostrophontodes* Lee & Huys, 2019, *Lobopleura*). However, as noted by George (2020), it is still not possible to satisfactorily resolve the relationships between those species remaining in *Laophontodes*.

Even with the addition of *L. volkerlehmanskii* sp. nov. as the 14^th^ species, we could not identify a derived feature to support the monophyletic status of *Laophontodes*. The apparent lack of shared morphological novelties within *Laophontodes* suggests that the taxon may represent the stem-lineage, retaining the derived characteristics of the common [*Ancorabolina – Bicorniphontodes – Laophontodes*]-ancestor, having “failed” to develop its own derived characters (Fig. 11). Whilst this is not uncommon (cf. Ax 1984; Sudhaus and Rehfeld 1992), the authors believe this might be the first evidence of a surviving stem-lineage in the Harpacticoida. It remains to be seen if future (molecular genetic) studies may support this hypothesis.

### Possible relations within *Laophontodes*

Phylogenetic relationships within *Laophontodes* cannot be resolved unambiguously. This is due to several reasons. For instance, the type material of many species is no longer available, preventing re-examination and comparison of most morphological characters. Moreover, as indicated by several authors (e.g., George and Gheerardyn 2015; George 2017, 2018, 2020; George et al. 2019; Lee and Huys 2019), species descriptions (especially, older publications) are fragmentary and of poor quality, precluding detailed comparisons between species. Nevertheless, such descriptions may be occasionally satisfactorily resolved, as shown below for character 2 (Table 3).

**Table 3.** List of 39 morphological characters used for the here presented phylogenetic analysis. In the second column, plesiomorphic states are set in square brackets. Columns 3–14: 1 = apomorphies; 0 = plesiomorphies; ? = no information available; **1** = supposed convergences. 0* = also apomorphic state present, due to intraspecific variability; explanation in the text.

**No.**	**Character [plesiomorphies in square brackets]/species**	*L. sarsi*	*L. scottorum*	*L. volkerlehmanskii* sp. nov.	*L. georgei*	*L. gertraudae*	*L. spongiosus*	*L. mourois*	*L. typicus*	*L. monsmaris*	*L. macclintocki*	*L. sabinegeorgeae*	*L. whitsoni*
**1**	A2 exopod represented by tiny seta [with 1 small, knob-like segment bearing 1 small seta]	1	1	1	1	1	1	1	1	1	1	1	1
**2**	A1 male 6-segmented, chirocer [7-segmented, subcirocer]	?	1	1	1	1	1?	1	1	1	0?	0	0
**3**	P4 female enp-2 lacking outer seta [seta present]	1	1	1	1	1	1	1	1	1	0	0	0*
**4**	P3exp-3 with at most 1 inner seta [with 2 setae]	1	1	1	1	1	1	1	1	0	**1**	0	0
**5**	P4exp-3 with at most 1 inner seta [with 2 setae]	1	1	1	1	1	1	1	1	0	**1**	0	0
**6**	P2 exp-2 lacking inner seta [seta present]	1	1	1	1	1	1	1	1	0	0	0	0
**7**	P3 exp-2 lacking inner seta [seta present]	1	1	1	1	1	1	1	1	0	0	0	0
**8**	P2exp-3 lacking inner seta [seta present]	1	1	1	1	1	1	0	0	0	0	0	0
**9**	P4exp-3 inner apical seta trimmed down, flexible [seta almost identical with outer apical element]	1	1	1	1	0	0	0	0	0	0	0	0
**10**	P2exp-3 inner apical seta trimmed down, flexible [seta almost identical with outer apical element]	1	1	1	0	0	0	0	0	0	0	0	0
**11**	P3exp-3 inner apical seta trimmed down, flexible [seta almost identical with outer apical element]	1	1	1	0	0	0	0	0	0	0	0	0
**12**	P2exp-3 down-trimmed inner apical seta bare [seta biplumose]	1	1	0	0	0	0	0	0	0	0	0	0
**13**	P3exp-3 down-trimmed inner apical seta bare [seta biplumose]	1	1	0	0	0	0	0	0	0	0	0	0
**14**	P4exp-3 down-trimmed inner apical seta bare [seta biplumose]	1	1	0	0	0	0	0	0	0	0	0	0
**15**	P5 female inner baseoendopodal seta of fish-bone aspect [seta bipinnate]	1	0	0	0	0	0	0	0	0	0	0	0
**16**	Body flattened [cylindrical]	0	1	0	0	0	0	0	0	0	0	0	0
**17**	Body somites laterally extended [not extended]	0	1	0	0	0	**1**	0	0	0	0	0	0
**18**	Anal operculum: posterior margin strongly serrated [with spinules]	0	0	1	0	0	0	0	0	0	0	0	0
**19**	Furcal tube pore long, displaced subapically [tube pore small, near furcal base]	0	0	1	0	0	0	0	0	0	0	0	0
**20**	P1 inner basal seta strongly diminished in size [of moderate length]	0	0	1	0	0	0	0	0	0	0	0	0
**21**	P1 outer basal seta with STE [lacking STE]	0	0	1	0	0	0	0	0	0	0	0	0
**22**	P1exp-1 outer seta with STE [lacking STE]	0	0	1	0	0	0	0	0	0	0	0	0
**23**	P5 male exopod: subapical outer seta with STE [lacking STE]	0	0	1	0	0	0	0	0	0	0	0	0
**24**	P1enp-2 apical long seta lost geniculation [seta geniculated]	0	0	0	1	0	0	0	0	0	0	0	0
**25**	P4 endopod 1 segmented [2-segmented]	0	0	0	1	0	0	0	0	0	0	0	0
**26**	P4enp-2 lacking inner seta [seta present]	0	0	0	0	1	0	0	0	0	0	0	0*
**27**	Abdominal somites except telson dorsally with H-like cuticular structures [such structures absent]	0	0	0	0	0	1	0	0	0	0	0	0
**28**	P2exp-3 innerapicalseta bare [seta biplumose]	0	0	0	0	0	0	1	1	0	0	0	0
**29**	P3 male enp-3 bulged out on its inner margin [margin straight]	0	0	0	0	0	0	1	0	0	0	0	0
**30**	Mxp lacking tiny seta accompanying claw [tiny seta present]	0	0	0	?	0	0	0	1	0	1	0	0
**31**	Mxp extremely strengthened [mxp of moderate size]	0	0	0	0	0	0	0	0	1	0	0	0
**32**	Telson overlapped by previous somite [not overlapped]	0	0	0	0	0	0	0	0	1	0	0	0
**33**	Rostrum frontally with tuft of long setules [no setular tuft]	0	0	0	0	0	0	0	0	0	0	1	0
**34**	Abdominal somites except telson dorsally with paired cuticular longitudinal ridges [such ridges absent]	0	0	0	0	0	0	0	0	0	0	1	0
**35**	Abdominal somites except telson dorsally with pairs of long tube pores [paired tube pores, if present, small]	0	0	0	0	0	0	0	0	0	0	1	0
**36**	FR mid-laterally with accessory long tube pore [lacking accessory tube pore]	0	0	0	0	0	0	0	0	0	0	1	0
**37**	FR setae I and II displaced subapically [arising mid-laterally]	0	0	0	0	0	0	0	0	0	0	1	0
**38**	P5 male exopod: proximal lateral seta with STE [lacking STE]	0	0	0	0	0	0	0	0	0	0	0	1
**39**	P5 male exopod: subapical inner seta with STE [lacking STE]	0	0	0	0	0	0	0	0	0	0	0	1

*Laophontodes antarcticus* and *L. propinquus species inquirenda* were excluded from the phylogenetic analysis presented herein, due to the fragmentary and imprecise descriptions by Brady (1918 and 1910, respectively), and the absence of type material for re-examination.

Careful examination of the remaining 12 species revealed 38 morphological characters as phylogenetically relevant. They are listed in Table 3 (characters 2–39) and are discussed in detail below. Four out of the 38 apomorphies are considered to be convergent (4, 5, 17, 30; underlined in Fig. 11), and the remaining 34 characters as unambiguous. The result of this phylogenetic analysis is graphically summarised in Fig. 11.

A group of nine species within *Laophontodes* (Table 3) share two derived features:

2. Male A1 6-segmented, chirocer [7-segmented, subchirocer];
3. Female P4 endopod with outer seta/spine lost [outer seta/spine still present].

These species (*L. sarsi*, *L. scottorum*, *L. volkerlehmanskii* sp. nov., *L. georgei*, *L. gertraudae*, *L. spongiosus*, *L. mourois*, *L. typicus*, *L. monsmaris*; Fig. 11) have lost the penultimate segment of the male A1, which therefore changes from subchirocer to chirocer (character 2) (not yet confirmed for *L. sarsi*, because the males remain unknown). Only three species, namely *Laophontodes macclintocki*, *L. sabinegeorgeae*, and *L. whitsoni*, retain the plesiomorphic 7-segmented, subchirocer male A1. Additionally, the description by Schizas and Shirley (1994) for *L. spongiosus* is contradictory; they state that the male A1 is subchirocer, but describe only one segment after the geniculation, which characterises it as a chirocer A1. Therefore, in Table 3 character 2 is marked with “1?” for *L. spongiosus*.

The derived chirocer condition is hypothesised as synapomorphic for the nine species and this is supported by the concurrent appearance of character 3, viz. the loss of the outer element of the female P4 endopod. Although the reduction of setae/spines occurs frequently and often independently in Harpacticoida, their simultaneous loss alongside the loss of the penultimate segment in the male A1 in all nine species strongly supports its synapomorphic status.

Remarks on character 2: Recent detailed descriptions of the male A1 revealed the existence of a very small fourth antennular segment between the third and the swollen fifth segment in *Laophontodes* (e.g., George and Gheerardyn 2015; George 2018). This tiny segment – already known for other Ancorabolidae – was first documented by Conroy-Dalton (2004) in males of other Laophontodinae (*Lobopleura ambiducta* Conroy-Dalton, 2004 and *Probosciphontodes* Fiers, 1988). The detection of a fourth antennular segment lead subsequent authors to confirm the presence of a small/tiny fourth segment in *Ancorabolina* George, 2006 (George 2006; George and Tiltack 2009; Gheerardyn and George 2010), *Bicorniphontodes* (George and Gheerardyn 2015; George et al. 2019), and *Calypsophontodes* Gheerardyn & Lee, 2012 (Gheerardyn and Lee 2012). This fourth segment has been also overlooked in *Laophontodes* (e.g., Schizas and Shirley 1994; Arroyo et al. 2003). However, the redescription of several laophontodin species (e.g., *Bicorniphontodes bicornis*, *Laophontodes typicus*, *L. whitsoni*), the description of new species of *Laophontodes* (George and Gheerardyn 2015; George 2018), and re-examination of available material of *Laophontodes mourois* (George pers. obs.) proved both the existence of this segment and that it had previously gone unnoticed. Therefore, it can be assumed with some certainty that this reduced fourth segment is also present in the male A1 of *L. macclintocki* and *L. spongiosus*.

Remarks on character 3: As documented by George and Gheerardyn (2015), the female P4enp-2 in *L. whitsoni* apparently presents an intraspecific variability; three examined females had four setae – two apical, one outer and one inner (formula I:2:1) – , while other females lacked the outer seta (0:2:1) or even both lateral elements (0:2:0). Considering that the secondary development of a formerly deleted element is possible (cf. George 2020 and references therein) but rather improbable, we conclude that *L. whitsoni* originally bears all four elements in the P4enp-2 (I:2:1). The reduction of the outer or both lateral setae is seen here as a deviation that has occurred within the species. Therefore, *L. whitsoni* is not grouped with those nine taxa that share the synapomorphic loss of the outer seta (character 3). Similarly, although *L. gertraudae* also lacks the inner seta of P4enp-2 (character 26), a closer relationship with *L. whitsoni* cannot be presumed due to the rarity of this character in the latter species. Nonetheless, this intraspecific variation and potential relationships are indicated by an asterisk * in the respective fields in Table 3.

Further relationships between *Laophontodes macclintocki*, *L. sabinegeorgeae*, and *L. whitsoni* remain unsolved (Fig. 11). Whilst each of these species can be characterised by at least two autapomorphies (Table 3, Fig. 11), no derived characters have been found that might support any sister-group relationship.

Another four derived characters are shared by eight species (*L. sarsi*, *L. scottorum*, *L. volkerlehmanskii* sp. nov., *L. georgei*, *L. gertraudae*, *L. spongiosus*, *L. mourois*, *L. typicus*; Table 3, Fig. 11):

4. P3 exp-3 with at most 1 inner seta [with 2 inner setae];
5. P4 exp-3 with at most 1 inner seta [with 2 inner setae];
6. P2 exp-2 lacking inner seta [seta present];
7. P3 exp-2 lacking inner seta [seta present].

Two inner setae on P3exp-3 (character 4) and P4exp-3 (character 5) are present in *L. monsmaris*, *L. sabinegeorgeae*, and *L. whitsoni*, while one inner seta was lost in the P3 and P4exp-3 of the remaining *Laophontodes* species. This is seen as the derived state and thus as synapomorphic for the respective species. An exception is *L. macclintocki*, in which an inner seta is lost in the P3 and P4exp-3. Unlike the other eight species in this group, *L. macclintocki* does not exhibit the synapomorphic state for characters 2 and 3, and therefore, the loss of the inner setae on the P3 and P3exp-3 in *L. macclintocki* can be assumed to be convergent. The alternative would be to assume that the apomorphic character of the chirocer A1 is the result of convergent development, which is far more implausible.

Furthermore, the eight species share derived characters 6 and 7, viz. the loss of the inner seta on P2 and P3 exp-2, respectively. Although we admit that characters 6 and 7 are rather weak because the reduction of elements may occur independently (see remarks on character 3), it is assumed that, together with characters 4 and 5, they constitute a set of deviations that were developed in a common ancestor of the eight species (Table 3, Fig. 11) and are thus interpreted as synapomorphies for them.

Six species share a single derived character (*L. sarsi*, *L. scottorum*, *L. volkerlehmanskii* sp. nov., *L. georgei*, *L. gertraudae*, *L. spongiosus*; Table 3, Fig. 11):

8. P2 exp-3 lacking inner seta [with 1 inner seta].

Among the above group of eight species, *Laophontodes typicus* and *L. mourois* (as well as all more basal species) show the plesiomorphic retention of an inner seta on the third exopodal segment of P2, whereas the remaining six species share its derived loss. This is seen here as synapomorphic for *L. spongiosus*, *L. gertraudae*, *L. georgei*, *L. volkerlehmanskii* sp. nov., *L. scottorum*, and *L. sarsi*.

Four species are characterised by the following putative synapomorphy (*L. sarsi*, *L. scottorum*, *L. volkerlehmanskii* sp. nov., *L. georgei*; Table 3, Fig. 11):

9. P4 exp-3 inner apical seta short, flexible [seta of normal length].

In the harpacticoid ground pattern, the two apical setae of P2–P4exp-3 are longer and more flexible than the outer spines of those segments, being of almost the same size. This state is retained in most *Laophontodes* species except for *Laophontodes sarsi*, *L. scottorum*, *L. volkerlehmanskii* sp. nov., and *L. georgei*. These species are characterised by a clearly diminished inner apical seta of the P2–P4exp-3, being much slenderer than the outer apical seta. This is interpreted as synapomorphic for these four species.

As with the other subgroups of the genus, relationships with those species excluded from the subgroup require further phylogenetic resolution (cf. interrogation marks in Fig. 11). The relationships of *Laophontodes spongiosus* and *L. gertraudae* with this last subgroup of four species, *L. sarsi*, *L. scottorum*, *L. volkerlehmanskii* sp. nov., and *L. georgei*, remain unclear, as no further apomorphic characters have been identified.

Three species – *L. sarsi*, *L. scottorum*, and *L. volkerlehmanskii* sp. nov. – share two further deviations (Table 3, Fig. 11):

10. P2 exp-3 inner apical seta short, flexible [seta of normal size];
11. P3 exp-3 inner apical seta short, flexible [seta of normal size].

In addition to the derived inner apical seta in the P4exp-3, *Laophontodes sarsi*, *L. scottorum*, and *L. volkerlehmanskii* sp. nov. exhibit a short, flexible seta on the P2 and P3exp-3, whilst *L. georgei* retains the normal-shaped inner apical setae. This is assumed as synapomorphic for the former species.

Finally, in *Laophontodes sarsi* and *L. scottorum* the inner apical seta of P2–P4 suffers a further deviation (Table 3, Fig. 11):

12. P2 exp-3 short inner apical seta bare [short inner apical seta biplumose];
13. P3 exp-3 short inner apical seta bare [short inner apical seta biplumose];
14. P4 exp-3 short inner apical seta bare [short inner apical seta biplumose].

In Harpacticoida, the inner and apical setae of P2–P4exp-3 are usually biplumose, which must be regarded as the plesiomorphic condition. Thus, the development of unarmoured, bare setae constitutes a deviation. Accordingly, the presence of the bare, short seta in P2–P4exp-3 is considered here as synapomorphic for *Laophontodes sarsi* and *L. scottorum*.

Remarks: The development of a bare inner apical seta in the P2exp-3 is also present in *L. typicus* and *L. mourois* (Table 3, character 28). Nevertheless, we assume that the loss of the setal ornamentation occurred independently in these species. Shortening of the inner apical setae presumably took place before the loss of their armour in *Laophontodes sarsi* and *L. scottorum*. This assumption is further supported by the fact that these short setae remain biplumose in *L. volkerlehmanskii* sp. nov.. In contrast, the length of the inner apical seta in the P2exp-3 of *L. typicus* and *L. mourois* is normal as in P3 and P4. A phylogenetic discussion of character 28 is given below.

The following characters, 15–39, listed in Table 3, characterise the different *Laophontodes* species. Direct comparison of characters was impossible for most species because of the lack of suitable type material. Consequently, the characterization of species by apomorphic characters is far from complete, with several species being characterised by just one potential apomorphy. Until further data are available, the current analysis provides sufficient information for an initial phylogenetic characterization of each species.

### Characterization of *Laophontodes* species

*Laophontodes sarsi*, character 15 (Table 3, Fig. 11): The common shape of the harpacticoid P5 baseoendopodal setae (including *Laophontodes*) is a bipinnate one, with the pinnae being distinct. This is considered the plesiomorphic state. In contrast, the pinnae are strengthened and fused to the seta in *L. sarsi*, giving a “fish-bone” appearance (George 2018). This shape is rarely seen in Harpacticoida, and it is considered to be derived, i.e., an apomorphic state.

*Laophontodes scottorum*, characters 16 and 17 (Table 3, Fig. 11): George (2020) considered a cylindrical, slender body – listed here as character 1 in Table 3 – as synapomorphic for *Ancorabolina*, *Bicorniphontodes*, and *Laophontodes*. According to George (2020), the plesiomorphic state consists of a fusiform body that tapers posteriorly. *Laophontodes scottorum* deviates from character 1 in presenting a robust, rather compact body somewhat dorsoventrally compressed (character 16) (T. Scott 1907; George 2018). Moreover, the body somites are laterally extended (character 17); these lateral extensions are reminiscent of epimeres in other harpacticoid taxa (T. Scott 1907; George 2018). However, *L. scottorum* exhibits the synapomorphic state for characters 2–14, clearly justifying its assignment to *Laophontodes*. Moreover, although its body shape does not fit the synapomorphic state for *Laophontodes*, it does not match the plesiomorphic condition either. Instead, it can be postulated that the body shape of *L. scottorum* represents a secondary deviation, in addition to Character 17. Both character states are considered autapomorphic for this species.

Character 17 is also present in *L. spongiosus* (cf. Schizas and Shirley 1994); however, it only shares this character and apomorphies 2–8 with *L. scottorum*, suggesting it branched off much earlier, not sharing apomorphies 9–14. Therefore, we assume that the lateral extension of the body somites occurred convergently in these two species.

*Laophontodes volkerlehmanskii* sp. nov., characters 18–23 (Table 3, Fig. 11): This newly described species presents a series of morphological differences compared to the remaining species of *Laophontodes*. To characterise it unambiguously, the following six autapomorphies were selected: Strong serration of the posterior margin of the anal operculum (character 18), which is unique within *Laophontodes* – with the anal operculum of almost all other species exhibiting a row of fine spinules; subapical displacement and elongation of the furcal tube pore (character 19), compared to the usually small furcal tube pore located on the outer anterior lateral margin of the ramus in most *Laophontodes* species; a strongly diminished inner seta on the P1 basis (character 20), which does not reach the endopod in *L. volkerlehmanskii* sp. nov., similarly contrasts to the synapomorphic condition for *Laophontodes* in which the inner basal seta of the P1 is of moderate length, usually reaching the endopod; development of STE on the P1 outer basal seta (character 21), the P1exp-1 outer spine (character 22), and the outer subapical seta of the male P5 exopod (character 23) are likewise exclusive derived features of *L. volkerlehmanskii* sp. nov., STE being rarely documented in Harpacticoida. In *Laophontodes*, only two species have been described possessing STE, namely *L. whitsoni* (characters 38, 39) (George and Gheerardyn 2015) and *L. volkerlehmanskii* sp. nov.. This has been confirmed by examination of various *Laophontodes* material (George pers. obs.). Thus, characters 18–23 are seen here as unambiguous autapomorphies of *Laophontodes volkerlehmanskii* sp. nov.

*Laophontodes georgei*, characters 24 and 25 (Table 3, Fig. 11): *L. georgei* was described as *L. norvegicus* George, 2018 by George (2018) and subsequently renamed by Lee and Huys (2019), with the illustrations provided by Sars (1908) as the holotype. It may be characterised by two deviations: Firstly, *L. georgei* has a long, non-geniculated apical seta on the P1enp-2 (character 24) (Sars 1908) compared to a long geniculated seta in all other *Laophontodes* species, as well as in *Ancorabolina* and *Bicorniphontodes*, which are considered closely related. Consequently, the geniculated seta is considered to be the plesiomorphic state. As an early harpacticoid description, being more than 100 years old (Sars 1908; as *L. typicus*), one might suspect this geniculation was overlooked. However, G.O. Sars was a keen observer, and in fact noted geniculated setae on the P1exp-3 of *L. georgei*. Thus, there is no reason to assume that he had overlooked the geniculation in the apical seta of P1enp-2. It is therefore concluded that in *L. georgei* the P1enp-2 apical seta lost the geniculation, resulting in an autapomorphic character for that species.

Moreover, *L. georgei* is the only *Laophontodes* species that exhibits a 1-segmented P4 endopod (Sars 1908; George 2018) (character 25). This reduction of the enp-1 is interpreted as autapomorphic of the species.

*Laophontodes gertraudae*, character 26 (Table 3, Fig. 11): All species of *Laophontodes*, except for *L. gertraudae*, bear a P4enp-2 with 1 inner seta; only *L. gertraudae* lacks it (George 2018; but see discussion on character 3). This is regarded as an autapomorphy for the species.

*Laophontodes spongiosus*, characters 17 and 27 (Table 3, Fig. 11): *L. spongiosus* has three derived characters that are pooled as one autapomorphy (character 27): the abdominal somites except the telson are characterised by H-like cuticular dorsal structures (Schizas and Shirley 1994). Such structures are unique within *Laophontodes*. They are reminiscent of similar structures found in *Paralaophontodes* (Lang 1965; Fiers 1986; George 2017), but as shown by George (2020), no closer relationship between *Paralaophontodes* and *L. spongiosus* exists. So, although these derived features appear to be convergent for the two taxa, they are considered autapomorphic for the latter.

In addition to character 27, another deviation discussed here is interpreted as convergent (character 17, cf. *L. scottorum*).

*Laophontodes mourois–L. typicus*-group, character 28 (Table 3, Fig. 11): The transformation of pinnate/plumose setae into bare elements has been discussed above (character 12). The rather ancestral inner apical seta in the P2–P4exp-3 of *Laophontodes* is biplumose, as observed in, for example, *L. gertraudae*, *L. monsmaris*, *L. spongiosus*, and *L. whitsoni*. Within the genus, however, two developmental directions were detected. The first is the reduction in length of the inner apical setae, followed by a subsequent loss of ornamentation (characters 9–11; 12–14); this is seen in *L. georgei* (on the P2), and in *L. volkerlehmanskii* sp. nov., *L. scottorum*, and *L. sarsi* (on P2–P4), and has been discussed above. A second developmental direction is seen in *Laophontodes mourois* and *L. typicus*, in which the length of the inner apical seta of the P2exp-3 is normal, but has lost it ornamentation. This derived state is considered as synapomorphic for *Laophontodes mourois* and *L. typicus*.

*Laophontodes mourois*, character 29 (Table 3, Fig. 11): Based on the description of Arroyo et al. (2003), *L. mourois* has one autapomorphy: The male P3enp-3 shows a rounded inner margin (character 29) compared to other *Laophontodes* males with a straight inner margin.

*Laophontodes typicus*, character 30 (Table 3, Fig. 11): *L. typicus* does not present any exclusive morphological deviations. Compared with other *Laophontodes* species, *L. typicus* seems to retain most plesiomorphic character states. Only two deviations have been observed in the species, characters 28 and 30, and these are shared with other congeners. Of these, character 28 supports a sister-group-relationship with *L. mourois* (see above). In contrast, character 30 – the lack of the minute seta accompanying the maxillipedal claw – , whilst also found in *L. macclintocki* (see below), is thought to be the result of convergence: *L. macclintocki* lacks the apomorphic state of character 28, but exhibits apomorphies 4 and 5 (see below), which are not seen in *L. typicus*. Therefore character 30 is regarded as autapomorphic for *L. typicus*.

According to the description of Sars (1908), no such minute seta is present in the maxillipedal claw of *L. georgei*. Future examination may reveal if this is true or if the seta was overlooked by Sars (1908).

*Laophontodes monsmaris*, characters 31, 32 (Table 3, Fig. 11): This species exhibits two autapomorphic characters, which are unique not only within *Laophontodes* but also in the Laophontodinae: the maxilliped is extremely elongated and strengthened (character 31), and the penultimate abdominal somite overlaps the telson (character 32) (George 2018). Because of these autapomorphies a phylogenetic characterization of *L. monsmaris* is unambiguous.

*Laophontodes macclintocki*, characters 4, 5, 30 (Table 3, Fig. 11): The convergent loss of 1 inner seta in P3 and P4exp-3 (characters 4 and 5) has been discussed above. In addition, *L. macclintocki* shares one further (convergent) deviation with *L. typicus*, viz. the loss of the tiny seta accompanying the maxillipedal claw (character 30). As stated by George (2018), the loss of this seta must be considered with care, since it has been overlooked in species descriptions. However, with respect to *L. macclintocki* we trust in the description of Schizas and Shirley (1994), who noted this tiny seta in *L. spongiosus* in the same publication and are therefore unlike to have missed it in *L. macclintocki*. As discussed above, we hypothesise that the loss of this seta is autapomorphic for *L. macclintocki* and that its absence in *L. typicus* is the result of convergence.

*Laophontodes sabinegeorgeae*, characters 33–37 (Table 3, Fig. 11): *L. sabinegeorgeae* may be unambiguously characterised by several derived characters (cf. George 2018). The species exclusively presents a tuft of long setules on the front of the rostrum (character 33); the presence of paired longitudinal cuticular ridges on the abdominal somites except the telson (character 34); the development of paired, remarkably long tube pores on the abdominal somites except the telson (character 35); a long tube pore arising mid-laterally on the FR (character 36), in addition to a small anterior tube pore found on the FR in other *Laophontodes* species, and, finally, the subapical displacement of furcal setae I and II (character 37).

Remarks on character 37: According to George et al. (2019) and Lee and Huys (2019), furcal setae I and II in species of *Laophontodes* are positioned in the distal half of the furcal rami, close to the centre line. This is the case in nine species, *L. georgei*, *L. gertraudae*, *L. monsmaris*, *L. mourois*, *L. sarsi*, *L. scottorum*, *L. typicus*, *L. volkerlehmanskii* sp. nov., and *L. whitsoni* (cf. Sars 1908; Arroyo et al. 2003; George and Gheerardyn 2015; George 2018; present contribution, Fig. 5A). However, a trend towards the apical displacement of setae I and II can be noted: in *L. macclintocki* and *L. spongiosus* they are displaced distally but still positioned on the outer lateral margin of the FR; in *L. sabinegeorgeae* they are almost in the subapical margin of the FR (George and Gheerardyn 2015). This latter position also resembles the derived condition as found in *Bicorniphontodes* (cf. George et al. 2019). The subapical position of furcal setae I and II in *L. sabinegeorgeae* is unique in *Laophontodes*.

*Laophontodes whitsoni*, characters 38 and 39 (Table 3, Fig. 11): *L. whitsoni* is the first species that branches off in the cladogram presented in Fig. 11. It presents two deviations regarding the male P5: both the proximal outer seta (character 38) and the subapical inner seta (character 39) present STE (George and Gheerardyn 2015) which are absent in all remaining *Laophontodes* species.

Remarks: Five species (*L. whitsoni*, *L. sabinegeorgeae*, *L. typicus*, *L. mourois*, and *L. scottorum*) present a further character that must be regarded as deviation, that is the development of fine longitudinal ripples dorsally on the pedigerous somites bearing the P2–P4 (Arroyo et al. 2003; George and Gheerardyn 2015; George 2018). Such ripples may even be seen on the remaining pedigerous somites as well as on the abdominal somites (except telson). However, if this character state is to be considered as synapomorphic for the above species, this would demote characters 2–14 to convergences (Table 3), which would be less parsimonious. Furthermore, while the absence of such ripples is confirmed for *L. gertraudae*, *L. monsmaris*, and *L. volkerlehmanskii* sp. nov. (George 2018; present contribution), it is still not known if they occur in *L. antarcticus*, *L. georgei*, *L. macclintocki*, *L. propinquus sp. inquirenda*, and *L. spongiosus*. Consequently, it was not possible to include this character in the here presented study.

## Summary and conclusion

The description of *Laophontodes volkerlehmanskii* sp. nov. facilitated an attempt to characterise the genus *Laophontodes* and to elucidate the phylogenetic relationships within the taxon. Careful comparison of 39 morphological characters led to the conclusion that *Laophontodes* cannot be characterised by any autapomorphies. Instead, it seems to reflect the stem-lineage of a monophylum comprised of *Ancorabolina*, *Bicorniphontodes*, and *Laophontodes*. While *Ancorabolina* and *Bicorniphontodes* can be characterised as monophyla and furthermore present a sister-group relationship (George 2020), *Laophontodes* retains the characters of the common ancestor, without having developed unique deviations that might be considered as synapomorphies of species assigned to the genus.

Similarly, discrimination of the 12 *Laophontodes* species examined here (*L. antarcticus* and *L. propinquus* excluded) is ambiguous. Most characters refer to the reduction of single setae or spines, which happens often and independently in harpacticoid species. Moreover, several features presumed to be derived, such as the development of fine dorsal cuticular ripples on the pedigerous somites P2–P4, or the lateral extension of the body somites, seem to be distributed quite heterogeneously amongst the species. Finally, many descriptions of *Laophontodes* species are incomplete or of poor quality, and the respective type material is no longer available. Those conditions have inhibited the comparison of all the morphological characters that may be otherwise of phylogenetic relevance.

Nonetheless, each of the *Laophontodes* species can be characterised by certain derived characters, even if convergence has to be assumed for some of them. Thus, the phylogenetic analysis undertaken provides insights into the phylogenetic relationships of and within *Laophontodes* and serves as the base for ongoing research.

## Supplementary Material

XML Treatment for Laophontodes

XML Treatment for Laophontodes volkerlehmanskii

## Footnotes

L. antarcticus Brady, 1918 and L. propinquus Brady, 1910 excluded
